# OCT, Triple H oder doch etwas anderes?

**DOI:** 10.1007/s00108-023-01559-1

**Published:** 2023-07-05

**Authors:** Anna Heinen, Rolf Erlebach, Claudia Schrimpf, Marco Bonani, Christoph C. Ganter, Sascha David, Rea Andermatt

**Affiliations:** 1 Institut für Intensivmedizin, Universitätsspital Zürich, Rämistr. 100, 8091 Zürich, Schweiz; 2 Klinik für Gefässchirurgie, Universitätsspital Zürich, Rämistr. 100, 8091 Zürich, Schweiz; 3 Klinik für Nephrologie, Universitätsspital Zürich, Rämistr. 100, 8091 Zürich, Schweiz

**Keywords:** Leberversagen, Ammoniak, Dialyse, Hämofilter, Cytosorb, Liver failure, Ammonia, Dialysis, Hemofilter, Cytosorb

## Abstract

Die Hyperammonämie ist ein lebensbedrohliches Krankheitsbild, dessen Prognose von einer raschen Senkung des Ammoniaks abhängt. Ist eine hepatische Ursache ausgeschlossen, ist die Differenzialdiagnose breit und umfasst auch im Erwachsenenalter hereditäre Stoffwechselerkrankungen. Hier beschreiben wir den Fall einer 25-jährigen Patientin mit einer schweren, therapierefraktären Hyperammonämie und betonen die Relevanz der extrakorporalen Elimination des Ammoniaks.

## Abstract (en)

Hyperammonemia is a life-threatening condition, the prognosis of which depends on a rapid reduction of ammonia. If a hepatic cause is excluded, the differential diagnosis is broad and even in adulthood includes hereditary metabolic diseases. Here, the case of a 25-year-old female patient with severe hyperammonemia refractory to standard therapy is described and the relevance of extracorporeal elimination of ammonia emphasized.

## Anamnese

Die Aufnahme der 25-jährigen Patientin erfolgte nach Out-of-hospital-Reanimation bei hypoxisch bedingtem Kreislaufstillstand nach Opiatintoxikation. Nach 20-minütiger Laienreanimation zeigte sich bei Eintreffen des Rettungsdiensts eine Asystolie. Nach weiteren 7 min Reanimation kam es zur Rückkehr des Spontankreislaufs. Akten- und fremdanamnestisch litt die Patientin unter psychiatrischen Vorerkrankungen (soziale Phobie, Dysthymie, Panikattacken) und einem Substanzabusus (Alkohol, Benzodiazepine).

## Befunde und Diagnostik

Bei Aufnahme auf der Intensivstation präsentierte sich die intubierte und mechanisch beatmete Patientin im Schock mit „mottling“ und kalter Peripherie. Die klinische Untersuchung war ansonsten unauffällig. Zur Aufrechterhaltung eines suffizienten Mitteldrucks waren hohe Dosen Noradrenalin notwendig.

Die Blutgasuntersuchung zeigte eine schwere Laktatazidose (pH 6,83, HCO_3_ 11,6 mmol/l, pCO_2_ 5,21 kPa, Laktat 14,2 mmol/l). Es fanden sich erhöhte Transaminasen, eine Koagulopathie (Spontan-INR 2,8, Faktor V < 10 %) sowie ein mäßig erhöhtes Ammoniak (NH_3_) um 100 μmol/l. Eine akute Virushepatitis wurde ausgeschlossen. Die toxikologische Untersuchung fiel für Alkohol und Benzodiazepine (Dauermedikation) positiv aus. Paracetamol war negativ. Das Coeruloplasmin war tief, die Leberperfusion regelrecht.

Konsekutiv wurde ein akutes Leberversagen bei ischämischer Hepatitis im Rahmen des Kreislaufstillstands diagnostiziert. Es erfolgten eine Therapie mit N‑Acetylcystein nach Prescott-Schema und eine zielgerichtete Substitution von Gerinnungsprodukten.

Der Aufwachversuch nach 24 h verlief erfreulich mit Beantwortung von geschlossenen Fragen und gezielter Bewegung der Extremitäten.

**Figure 1:**
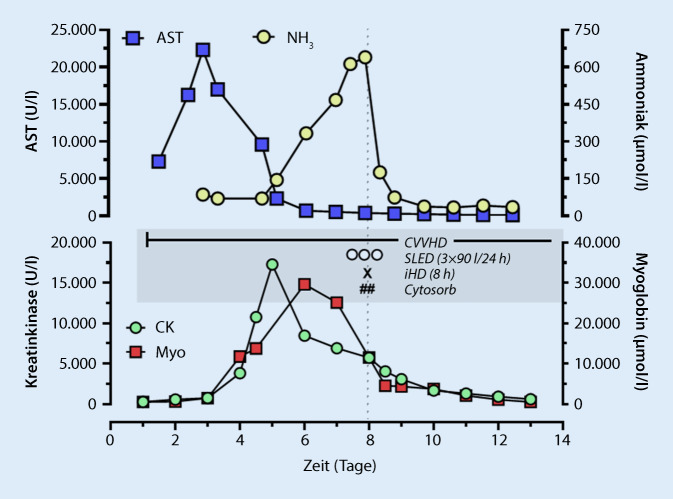

Nach initialer Stabilisierung verschlechterte sich die Patientin erneut mit Entwicklung einer Oligurie und einer metabolischen Azidose, sodass eine kontinuierliche venovenöse Hämodialyse (CVVHD) mit regionaler Citratantikoagulation (Fresenius, MultifiltratePRO, Q_B_ 200 ml/min, Q_D_ 2000 ml/h) erfolgte. Im weiteren Verlauf präsentierte sich die Patientin hoch-inflammiert im distributiven Schock mit extensivem Katecholaminbedarf (bis maximal 1,3 μg/kg pro min Noradrenalin, 0,03 U/min Vasopressin, 200 μg/min Dobutamin) und einem hohen Volumenbedarf. Als ursächlich hierfür fanden sich eine Aspirationspneumonie und eine akute ischämische Pankreatitis. Es erfolgte eine empirische antiinfektive Therapie mit Meropenem, Vancomycin und Caspofungin. Als weitere Komplikation entwickelte die Patientin ein fulminantes Kompartmentsyndrom beider Unterschenkel mit Bedarf einer notfallmäßigen Fasziotomie. In den folgenden Tagen zeigte sich unter supportiver Therapie eine kontinuierliche hämodynamische Stabilisierung. Dennoch zeigte sich nun trotz laufender CVVHD ein rasch progredient steigendes NH_3_ bis zu einem Maximalwert von 640 μmol/l (Abb. 1). Zu diesem Zeitpunkt hatte sich die Lebersynthese bereits deutlich verbessert (INR 1,4), sodass eine rein hepatische Ätiologie der Hyperammonämie (HA) unwahrscheinlich erschien. Klinisch fanden sich keine Hinweise für eine gastrointestinale Blutung und es bestand weder eine exogen erhöhte Proteinzufuhr noch eine anatomische Variante im Sinne von portosystemischen Shunts. Bei der Durchsicht der Medikamente fand sich kein Medikament, welches mit der Entwicklung einer HA assoziiert war. Weiter konnte kein ureaseproduzierendes Bakterium nachgewiesen werden, insbesondere auch keine gezielt gesuchten Ureaplasmen. Weiter konnte eine Infektion mit *Mycoplasma hominis* ausgeschlossen werden, welche über die Depletion von Arginin, einem notwendigen Kofaktor im Harnstoffzyklus, zu einer HA führt. In der Folge wurden metabolische Krankheiten mit reduzierter Clearance von NH_3_ mit später Manifestation im Erwachsenenalter diskutiert. Zum Ausschluss wurde eine Aminosäurenbestimmung im Serum durchgeführt. Hier zeigten sich ein deutlich erhöhtes Ornithin und Glutamin bei jedoch nur minimal erhöhtem Arginin und normwertigem Citrullin. Konsekutiv konnte ein Ornithin-Transcarbamylase(OCT)-Mangel ausgeschlossen werden. Bei der nachgewiesenen Hyperornithinämie wurde ein Triple-H-Syndrom diskutiert, jedoch fehlte hier ein erhöhtes Homocitrullin im Urin, sodass die Trias des Triple-H-Syndroms nicht erfüllt war. Auf genetische Abklärungen bezüglich dieser Erkrankungen wurde bei nicht erfüllten Kriterien verzichtet.

## Diagnose

Retrospektiv wurde in Zusammenschau der Befunde die schwere HA im Rahmen des massiven Proteinanfalls durch die Rhabdomyolyse und im Kontext der eingeschränkten Leberfunktion interpretiert.

## Therapie und Verlauf

Aufgrund der drohenden Gefahr eines Hirnödems bei dieser grotesken NH_3_-Erhöhung erfolgte eine umgehende Intensivierung der extrakorporalen Elimination durch 1) eine Dosissteigerung der CVVHD und 2) eine zusätzliche Cytosorb-Adsorption sowie parallel 3) ein hoch dosiertes *Slow-extended-dialysis*(SLED)-Regime (3 × 90 l in 24 h; FMC, Genius) über einen zweiten Shaldon-Katheter. Bei zunächst auch hierunter nicht fallendem NH_3_ veranlassten wir zudem eine verlängerte intermittierende Hämodialyse (8 h, Q_B_ 300, Q_D_ 500) über einen dritten Katheter. Der Verlauf des NH_3_ unter Therapie ist in Abb. 1 dargestellt. Parallel erfolgten zur Verminderung der intestinalen NH_3_-Produktion die Therapie mit Lactulose und Rifaximin sowie eine Stimulierung der Harnstoffsynthese in der Leber mit Ornithinaspartat. Außerdem wurden Natriumbenzoat, Natriumphenylacetat und Arginin über 24 h appliziert, um den Harnstoffzyklus und alternative Stoffwechselwege zur NH_3_-Elimination zu stimulieren. Mittels Applikation von hoch dosierter Glukose intravenös und eines Stopps der exogenen Proteinzufuhr wurde eine anabole Stoffwechsellage angestrebt.

Letztendlich konnte unter all diesen Maßnahmen das NH_3_ gesenkt werden und blieb im Verlauf ohne Maßnahmen im Normbereich.

Erfreulicherweise konnte die Patientin am 13. Tag extubiert und bei Besserung des Delirs zügig auf die Normalstation und schließlich ohne neurologische Defizite in die Rehabilitationsklinik verlegt werden. Nach Rehabilitation und Prothesenversorgung konnte die Mobilität wiedererlangt werden und die Patientin nach Hause austreten.

## Diskussion

Eine HA kann durch den intrazellulären Anstieg von Glutamin in den Astrozyten zu einem zerebralen Ödem führen. Das Hirnödem kann rasch progredient sein und schließlich zu einer Herniation führen. Häufig weisen Überlebende bleibende neurologische Schäden auf [1].

Bei Erwachsenen gilt für NH_3_ ein Referenzwert von < 50 μmol/l. Bereits bei einem Wert von > 200 μmol/l sind extrakorporale Verfahren indiziert [2]. Die Prognose der HA ist abhängig von einer möglichst raschen Senkung des NH_3_. Mit Komplikationen durch schnell abfallendes NH_3_ ist nicht zurechnen [2].

Am häufigsten ist eine HA im Erwachsenenalter hepatisch bedingt. Die Differenzialdiagnose der nichthepatischen Ursachen ist jedoch wie oben dargestellt breit. Bezüglich der medikamentös induzierten HA gibt es gewisse Chemotherapien, die zu einem vermehrten Anfall von NH_3_ führen, auch Medikamente, die die Clearance von NH_3_ reduzieren. Als prominentes Beispiel führt Valproat über eine Inhibierung der Carbamoylphosphat-Synthetase und Entwicklung eines Carnitinmangels zu einer HA. Weitere Medikamente, die häufig mit HA assoziiert sind, sind Tacrolimus und Ciclosporin, aber auch Gabapentin, Salizylate und Ribavirin [3].

**Figure 2:**
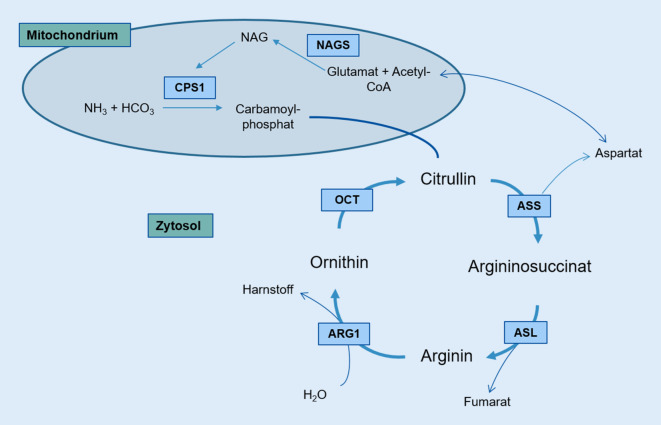

Hereditäre Erkrankungen des Harnstoffzyklus manifestieren sich meist im Kindesalter, können aber auch erst im Erwachsenenalter manifest werden [1]. Die häufigste Harnstoffzykluserkrankung, der OCT-Mangel, wird X‑chromosomal-rezessiv vererbt und weist Mutationen im OTC-Gen auf. Eine pränatale Diagnose ist in Familien mit bekannter Mutation möglich. Charakteristisch ist eine HA mit erhöhtem Ornithinserumspiegel und erniedrigtem Citrullin und Arginin. Die Orotatausscheidung im Urin ist erhöht. Diese Konstellation kann anhand der Abb. 2 gut nachvollzogen werden. Die Klinik des OCT-Mangels ist unspezifisch mit Nahrungsverweigerung, Lethargie, Apathie, Multiorganversagen, Muskelhypotonie und Thermolabilität.

Das seltene Triple-H-Syndrom kann sich auch erst im Erwachsenenalter manifestieren mit psychiatrischen Auffälligkeiten sowie einer Fleischvermeidung. Es beruht auf Mutationen im *SLC5A15*-Gen und wird autosomal-rezessiv vererbt [4]. Die Diagnosestellung beruht auf der typischen Trias aus Hyperornithinämie, HA und Homocitrullinurie und dann einer molekulargenetischen Bestätigung.

## Fazit für die Praxis

- Eine HA > 200 μmol/l ist aufgrund des hohen Risikos eines Hirnödems ein medizinischer Notfall. Die neurologische Prognose ist abhängig von einer möglichst raschen Senkung.
- Neben der supportiven Therapie mit Ornithinaspartat, Lactulose und Rifaximin sollten auch extrakorporale Eliminationsmöglichkeiten rasch eingeleitet werden.
- Die Ursachen der HA sind vielfältig. Bei HA und psychiatrischen Auffälligkeiten ist auch im Erwachsenenalter die Erstmanifestation einer Harnstoffzyklusstörung möglich.
